# Inducing Strong Light–Matter Coupling and Optical Anisotropy in Monolayer MoS_2_ with High Refractive Index Nanowire

**DOI:** 10.1021/acsami.2c07705

**Published:** 2022-06-28

**Authors:** Abde Mayeen Shafi, Faisal Ahmed, Henry A. Fernandez, Md Gius Uddin, Xiaoqi Cui, Susobhan Das, Yunyun Dai, Vladislav Khayrudinov, Hoon Hahn Yoon, Luojun Du, Zhipei Sun, Harri Lipsanen

**Affiliations:** † Department of Electronics and Nanoengineering, Aalto University, Tietotie 3, Espoo FI-02150, Finland; ‡ QTF Centre of Excellence, Department of Applied Physics, Aalto University, Aalto FI-00076, Finland

**Keywords:** MoS_2_, AlGaAs, mixed-dimensional heterostructure, electromagnetic field confinement, rotational symmetry breaking, light−matter interactions

## Abstract

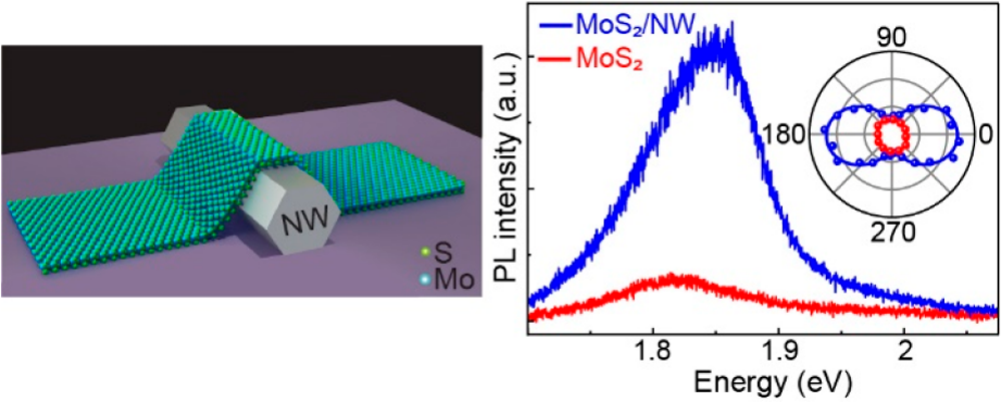

Mixed-dimensional heterostructures combine the merits of materials of different dimensions; therefore, they represent an advantageous scenario for numerous technological advances. Such an approach can be exploited to tune the physical properties of two-dimensional (2D) layered materials to create unprecedented possibilities for anisotropic and high-performance photonic and optoelectronic devices. Here, we report a new strategy to engineer the light–matter interaction and symmetry of monolayer MoS_2_ by integrating it with one-dimensional (1D) AlGaAs nanowire (NW). Our results show that the photoluminescence (PL) intensity of MoS_2_ increases strongly in the mixed-dimensional structure because of electromagnetic field confinement in the 1D high refractive index semiconducting NW. Interestingly, the 1D NW breaks the 3-fold rotational symmetry of MoS_2_, which leads to a strong optical anisotropy of up to ∼60%. Our mixed-dimensional heterostructure-based phototransistors benefit from this and exhibit an improved optoelectronic device performance with marked anisotropic photoresponse behavior. Compared with bare MoS_2_ devices, our MoS_2_/NW devices show ∼5 times enhanced detectivity and ∼3 times higher photoresponsivity. Our results of engineering light–matter interaction and symmetry breaking provide a simple route to induce enhanced and anisotropic functionalities in 2D materials.

## Introduction

Since the exfoliation of graphene, the unique electrical, optical, magnetic, and topological properties of two-dimensional (2D) van der Waals (vdW) materials have attracted significant interest and largely transformed the landscape of fundamental research and technological advances in physics, material sciences, and chemistry.^1−4^ Remarkably, the dangling-bond-free nature of 2D materials enables them to be integrated with non-2D materials (e.g., 0-, 1-, or 3-dimensional materials) through noncovalent interactions to form emerging mixed-dimensional vdW heterostructures.^5−7^ These heterostructures can combine the synergistic advantages of different dimensional materials, thus providing a more favorable platform than bare 2D materials for numerous advanced applications ranging from on-chip photodetectors to nanolasers.^8−13^

Usually, atomically thin 2D layered materials such as transition metal dichalcogenides (TMDCs) suffer from poor luminescence quantum yield because of defect-mediated nonradiative electron–hole recombination and a very short light–matter interaction length compared with the bulk crystals. These properties result in low-performance 2D TMDC-based optoelectronic devices.^14,15^ To date, a wide variety of approaches have been reported to enhance the light–matter interaction in 2D materials,^16,17^ e.g., by incorporating Fabry–Perot optical cavities,^18^ waveguides,^19^ plasmonic structures,^20^ and meta-surfaces.^21^ Among these techniques, plasmonic nanostructures made of noble metals (e.g., Ag and Au NWs) are the most advanced in modulating light–matter interactions in 2D materials. However, the structures and their fabrication processes are typically complex, suffer from metal-induced optical losses, and are incompatible to be integrated with semiconductor fabrication processes.^22,23^ Semiconductor nanowires (NW) with high refractive indices can provide a solution to avoid these issues and provide new opportunities to functionalize 2D materials.

Recently, III–V semiconducting one-dimensional (1D) NWs have emerged as promising candidates for various optoelectronic applications because of their direct band gap, simple and low-cost synthesis, high integration ability, and precise control in doping.^24−27^ As a III–V semiconducting material, AlGaAs NW possesses a high-refractive index and generates highly localized and strong optical fields within its 1D geometry. Furthermore, these NWs are suitable to be integrated with nanophotonic elements, optical circuits, or other dimensional materials because of their matured transfer and growth processes. Simple integration of 2D TMDCs with such 1D NWs can readily tailor the excitonic response of TMDCs by enabling strong light–matter interactions. Additionally, the hexagonal lattice structure of a monolayer TMDC has 3-fold (*C*_3_) rotational symmetry and inherently broken inversion symmetry.^28^ Typically, *C*_3_ symmetry of these materials can be broken by bending, applying strain, and reduction in dimensionality. Consequently, the materials manifest strong anisotropic vibrational, optical, and electrical responses.^29−32^

In this work, we report a new approach to engineer the light–matter interaction and symmetry of monolayer MoS_2_ by integrating it with 1D AlGaAs NW. Because of the strong electromagnetic (EM) field confinement in the NW, MoS_2_ photoluminescence (PL) increases significantly in the mixed-dimensional heterostructure compared with that in a bare MoS_2_ flake. Further, the mixed-dimensional heterostructure breaks the 3-fold rotational symmetry of 2D MoS_2_ explicitly, which leads to strong anisotropic optical responses. Additionally, we fabricate mixed-dimensional heterostructure-based phototransistors and benchmark their performance against bare MoS_2_ phototransistors. Compared with bare MoS_2_ devices, 2D MoS_2_/1D AlGaAs NW phototransistors show improved detectivity and photoresponsivity with marked anisotropic photoresponse due to enhanced light–matter interaction and symmetry breaking. Our findings establish mixed-dimensional heterostructures as a promising approach to realize strong and polarization-sensitive light–matter interactions in 2D materials for various photonic and optoelectronic applications.

## Results and Discussion

**Figure 1:**
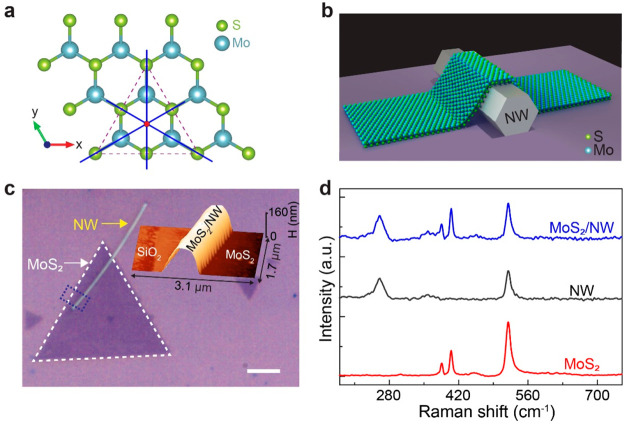
Our 2D MoS_2_/1D AlGaAs NW mixed-dimensional heterostructure. (a) Schematic of the crystal structure of monolayer 2H-MoS_2_. The blue color lines and red circle at the center of the triangle represent mirror reflection planes and the 3-fold rotational symmetry axis of the crystal, respectively. (b) Illustration of monolayer MoS_2_ transferred over a NW. (c) Optical image of a typical MoS_2_/NW sample. Scale bar: 5 μm. Inset shows an atomic force microscope (AFM) image of the heterostructure measured at the blue dashed rectangular area of the image. (d) Raman spectra of bare MoS_2_, a AlGaAs NW, and a MoS_2_/NW heterostructure.

The crystal structure of monolayer 2H-phase MoS_2_ is illustrated in Figure 1a, in which one layer of Mo atoms is sandwiched between two layers of S atoms to form a 2D hexagonal lattice structure. The unit cell of monolayer MoS_2_ is noncentrosymmetric and belongs to the nonpolar *D*_3h_ point group. In the monolayer MoS_2_ crystal, mirror reflection and 3-fold rotational symmetries are respected along the armchair direction. A schematic of the 2D MoS_2_/1D AlGaAs NW mixed-dimensional heterostructure is shown in Figure 1b. The chemical vapor deposition (CVD)-grown, monolayer MoS_2_ flakes are transferred onto NWs by wet transfer method (see Materials and Methods for details), as shown in the optical microscope image in Figure 1c. The typical diameter of the NWs used in this study is ∼80–150 nm. When monolayer MoS_2_ is transferred over a NW, the MoS_2_ layer covers the NW with a small suspended area close to both lower facets of NW. Figure 1d presents the typical room-temperature Raman spectra of 2D MoS_2_, a 1D AlGaAs NW, and a MoS_2_/AlGaAs mixed-dimensional heterostructure under 532 nm (∼2.33 eV) laser excitation. In the range of 250 cm^–1^ to 450 cm^–1^, two distinctive MoS_2_ Raman modes—in-plane E_2g_^1^ (∼385.50 cm^–1^) and out-of-plane A_1g_ (∼405 cm^–1^)—and the prominent AlGaAs NW mode—LO (∼260 cm^–1^)—are observed. These modes agree with the previous literature.^33−35^ The peak at ∼521 cm^–1^ corresponds to the Raman mode of the Si substrate.

**Figure 2:**
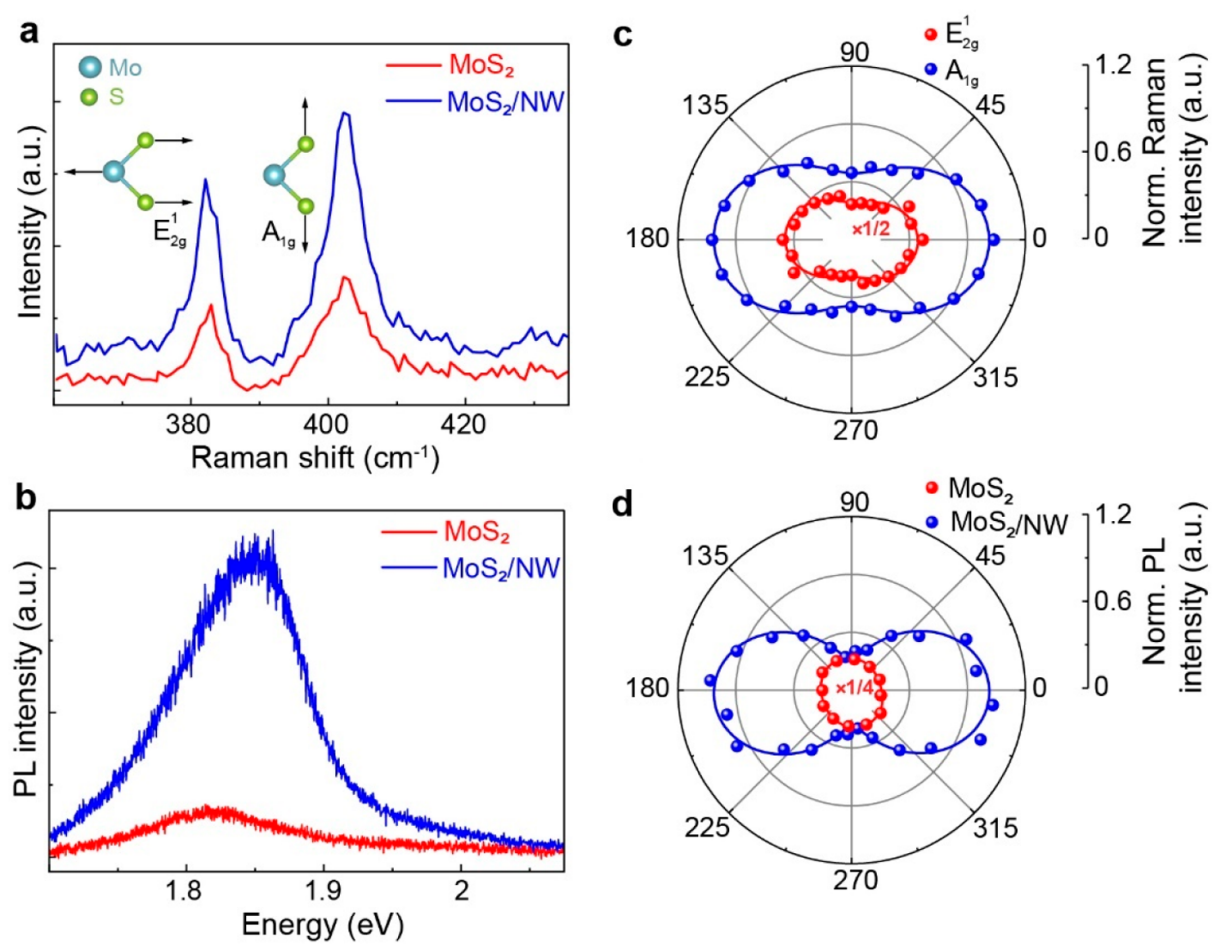
Enhanced and broken-symmetry-induced anisotropic optical response of mixed-dimensional heterostructures. (a) Raman spectra of a bare MoS_2_ flake and a MoS_2_/NW heterostructure at room temperature. Inset shows the schematic of the crystal structure with in-plane and out-of-plane Raman modes adjacent to the corresponding peaks. (b) Comparison of room temperature PL spectra of a bare MoS_2_ flake and a MoS_2_/NW heterostructure. (c) Variation of the E_2g_^1^ and A_1g_ mode normalized intensities in a MoS_2_/NW heterostructure at different polarization angles of the incident light. The polarization angle θ denotes the angle between the NW long axis and the polarization detection angle. (d) Anisotropy of PL in the MoS_2_/NW heterostructure and bare MoS_2_. Solid lines in (b) and (d) are fitted curves using a cos^2^θ function.

The enhanced light–matter interactions in the mixed-dimensional heterostructure were investigated by comparing the linear optical responses of the MoS_2_/NW heterostructure with bare MoS_2_ samples. In Figure 2a, both MoS_2_/NW heterostructure Raman modes match perfectly with those of monolayer MoS_2_, which indicates the intact quality of the transferred monolayer MoS_2_ onto NW. Interestingly, the Raman intensity from the MoS_2_/NW heterostructure increased by ∼3 times compared with the bare MoS_2_. In comparison with the Raman spectrum of bare MoS_2_ flake (red line), we observe a small peak shift of around −0.6 cm^–1^ from both E_2g_^1^ and A_1g_ modes of the MoS_2_/NW structure (blue line). This indicates a small strain and doping of MoS_2_ flake in the heterostructures.^36−39^ The estimated uniaxial local strain on MoS_2_ induced by a ∼ 100 nm diameter NW is ∼0.3% (see Section S1). Previously reported results show that the E_2g_^1^ Raman mode of MoS_2_ shifts by −1.7 cm^–1^ per percent of strain.^40^ The Raman peak shift due to strain in our samples is comparable. Therefore, the strain effect in our mixed-dimensional heterostructures is negligible.

Figure 2b shows the room temperature PL spectra of MoS_2_ with and without NW under 532 nm excitation. We observe a strong PL emission from bare MoS_2_ at 1.86 eV. The PL intensity from the MoS_2_/NW heterostructure increases remarkably by a factor of ∼9. We notice a small ∼25 meV blue shift of the MoS_2_ A-exciton peak from the heterostructure, which could be the reason for the reduced binding energy of the exciton due to an increased Coulomb screening of monolayer MoS_2_ induced by NW.^41^ It is pertinent to note that the uniaxial tensile strain usually causes redshift in PL.^40,42^ However, we observe a blue shift in the PL which implies that there is no influence of strain in enhancing the light–matter interaction in our mixed-dimensional heterostructures.

Furthermore, the polarization-dependent Raman intensity variations are shown in Figure 2c with a polar diagram, where the maximum and minimum intensity of the MoS_2_ Raman modes are recorded when the polarization of the incident linearly polarized pump light is parallel (θ = 0°) and perpendicular (θ = 90°) to the NW long axis, respectively. We define polarization angles as the difference between the polarizations of the linearly polarized pump light and the NW long axis. The degree of anisotropy^43^ [defined as, (*I*_max_ – *I*_min_)/(*I*_max_ + *I*_min_)] is calculated as ∼34% and 36% for modes E_2g_^1^ and A_1g_, respectively. A polar plot for comparing the polarization-dependent PL responses from bare MoS_2_ and MoS_2_/NW heterostructure samples is shown in Figure 2d. We do not observe any polarization angle dependency of PL emission from bare MoS_2_. In marked contrast, MoS_2_ shows a strong anisotropic PL response from the heterostructure. The PL intensity reaches its maximum (minimum) value when the excitation polarization is parallel (perpendicular) to the NW axis. The degree of MoS_2_ PL anisotropy from the heterostructure is calculated as ∼60%. The effective dimension of MoS_2_ is reduced from 2D to 1D at the heterostructure region, which breaks the *C*_*3*_ rotational symmetry of MoS_2_ and leads to strong anisotropy in the PL response.^30^ The PL response from monolayer MoS_2_ is much stronger than the Raman signals. This could be the reason for such a difference in the enhancement factor and degree of anisotropy between the Raman and PL of MoS_2_/NW.

The Raman and PL enhancement factor (*EF*), defined as the intensity ratio of signals measured in MoS_2_/NW and MoS_2_, depends on the NW diameter. In this work, the diameter of NWs varies between 80 and 150 nm (see Figure S1a). In general, *EF* increases with an increasing NW diameter, which offers a larger interfacial area in the heterostructures. We observe the highest Raman and PL *EF* of ∼3 and ∼9, respectively, with NWs of ∼150 nm diameter. The MoS_2_ Raman and PL *EF* as a function of NW diameters is shown in Figure S1b.

**Figure 3:**
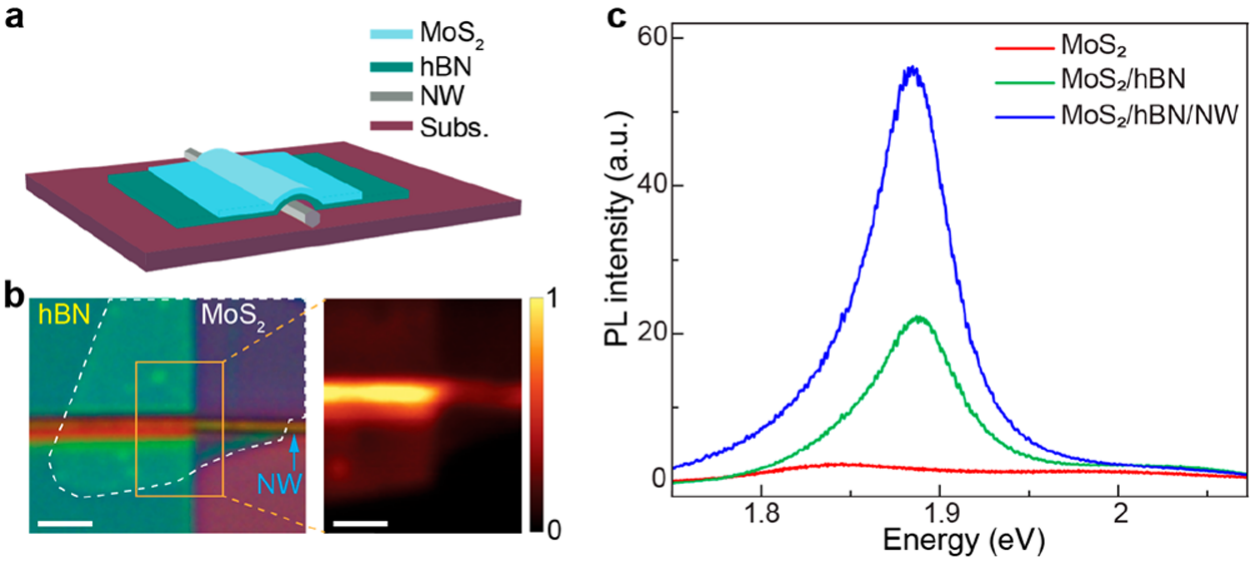
PL responses of a hBN intercalated MoS_2_/hBN/NW heterostructure. (a) Schematic of the heterostructure. (b) Optical image (left panel) of the sample, where the white dashed line indicates the area of monolayer MoS_2_ partially covering the hBN layer and a NW. Scale bar: 2 μm. The PL intensity mapping (right panel) is taken from the area marked in an orange rectangular box in the optical image. Scale bar: 1 μm. (c) PL spectra from bare MoS_2_, MoS_2_/hBN, and MoS_2_/hBN/NW regions.

The enhanced PL emission from our heterostructures may arise from different reasons, e.g., charge transfer,^41,44,45^ and optical field confinement in NW. On the basis of the Al composition of ∼30% (±5%) in AlGaAs NW in this study,^43^ the MoS_2_ and NW energy diagrams suggest a type-II band alignment at the heterostructure interface (see Figure S8a). Therefore, quenching of the MoS_2_ PL from the heterostructure is expected.^46^ In contrast, our results show enhancement in the MoS_2_ PL response from the heterostructure. We fabricated a mixed-dimensional sample where a multilayer (∼11 nm) hexagonal boron nitride (hBN) flake is inserted between the monolayer MoS_2_ and AlGaAs NW to determine the reason behind the enhanced optical properties in the MoS_2_/NW heterostructure and rule out the charge transfer mechanism. The insulating hBN would prevent any possible charge transfer in the heterostructure.^47,48^Figure 3a,b shows the schematic and the optical image of the hBN intercalated mixed-dimensional heterostructure, respectively. A PL mapping taken from the highlighted area of the optical image shows maximum emission from the MoS_2_/hBN/NW region. The PL responses at different locations are presented in Figure 3c. A blue shift of ∼50 meV in MoS_2_ PL from both the MoS_2_/hBN and MoS_2_/hBN/NW regions compared with that in the bare MoS_2_ (on SiO_2_/Si) is attributed to the hBN substrate effect.^39^ Compared with SiO_2_, hBN provides an atomically clean interface with 2D materials because of the low-density of charged impurities. Therefore, the enhancement in optical and electrical characteristics of 2D materials on hBN is expected. The PL intensity of MoS_2_ on hBN increases by ∼9 times compared with bare MoS_2_. Surprisingly, the PL intensity from the MoS_2_/hBN/NW heterostructure increases further by ∼3 times compared with that in the MoS_2_/hBN heterostructure. This confirms that the charge transfer phenomenon is not responsible for the enhanced optical properties from our mixed-dimensional heterostructures.

**Figure 4:**
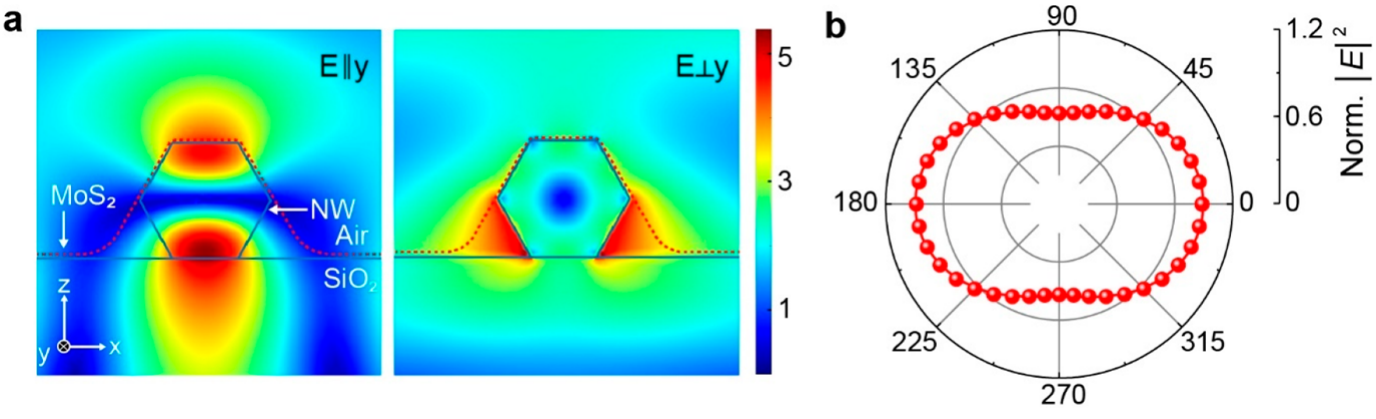
Numerical simulations of EM field distribution in mixed-dimensional heterostructure under 532 nm excitation. (a) EM field confinement around MoS_2_/NW heterostructure for excitation polarization parallel (left panel) and perpendicular (right panel) to the NW axis (in this case, *y* axis). (b) Variation of the EM field density at the top of the NW for different excitation polarization states.

We perform numerical simulations employing finite element modeling to understand the origin of the enhanced optical response in our mixed-dimensional heterostructure. For the simulation, we consider a monolayer MoS_2_ with a refractive index (*n*) of ∼3 for a 532 nm laser excitation^49^ is placed on a single hexagonal AlGaAs NW (*n* = 3.5)^50^ of 100 nm diameter. The heterostructure resides on a SiO_2_/Si substrate with a 285 nm thick SiO_2_ capping layer (*n* = 1.45). The model structure is illuminated normally with a linearly polarized excitation at a wavelength of 532 nm. As shown in Figure 4a, our results reveal strong EM field confinement in the NW, because of its high aspect ratio, and a large refractive index contrast compared with the surrounding media. Interestingly, when the excitation polarization direction is parallel to the NW axis, the confinement occurs at the top and bottom surfaces of the NW. In contrast, for the perpendicular configuration, the confinement becomes localized only near the bottom sides of the NW. The strong field confinement at the top of the NW results in an enhanced light–matter interaction in MoS_2_, which leads to a higher exciton excitation rate in the heterostructure region than in bare MoS_2_. Figure 4b summarizes the strength of the EM field confined only at the top of NW under different excitation polarization. Because of the anisotropy of the confined field and the 3-fold rotational symmetry breaking in monolayer MoS_2_, the strong anisotropy of the MoS_2_ PL in the mixed-dimensional heterostructure is enabled.

**Figure 5:**
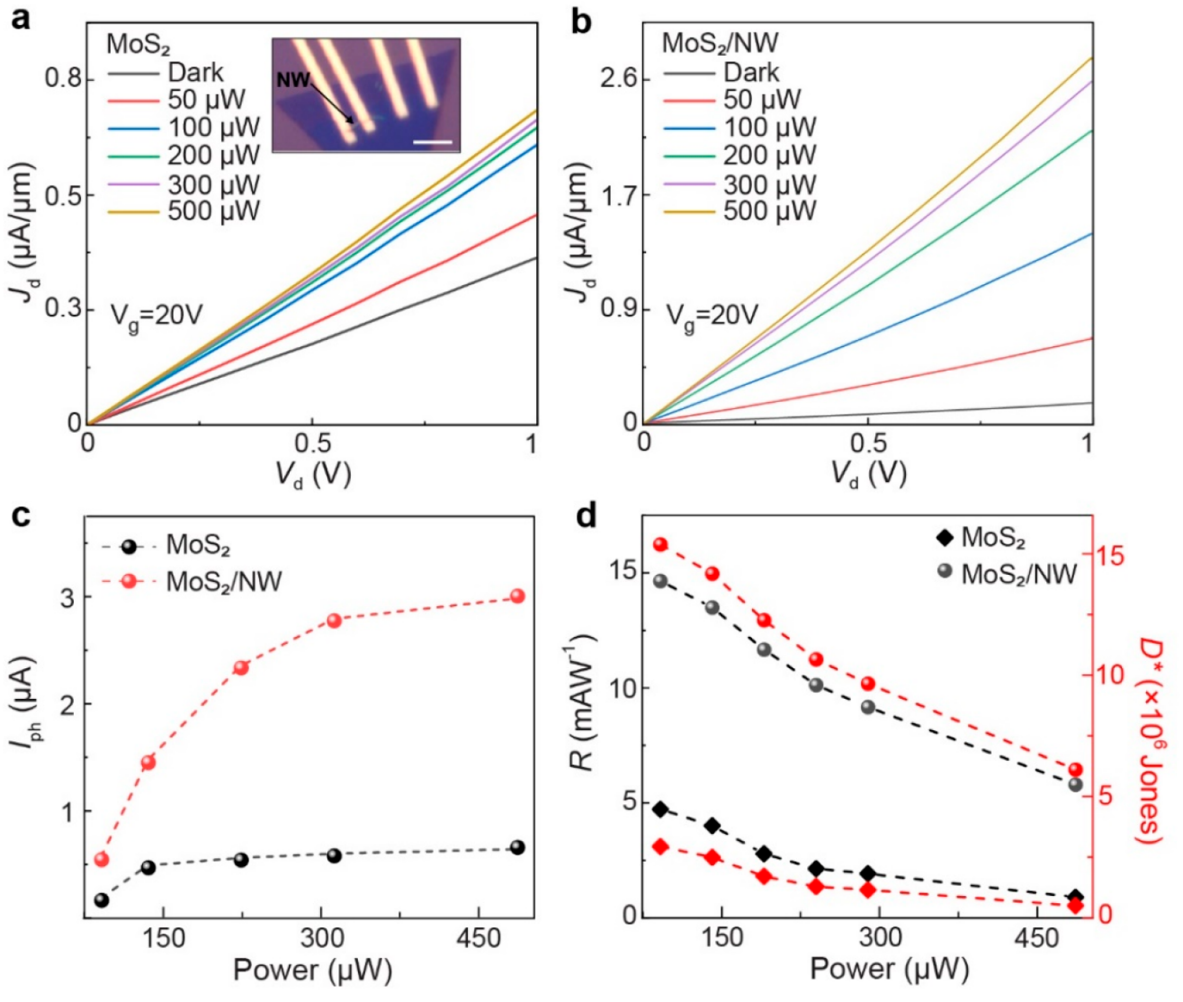
Improved performance of a mixed-dimensional MoS_2_/NW photodetector. (a,b) *I*_d_–*V*_d_ characteristics of MoS_2_ and MoS_2_/NW FETs in the dark and 532 nm laser illumination. An optical image of the devices is presented in the inset of (a). Scale bar: 2 μm. (c) Incident laser power-dependent photocurrent in the devices. (d) Comparison of responsivity and specific detectivity of the photodetectors as a function of incident laser power.

After understanding the anisotropic and enhanced light–matter interactions in the MoS_2_/NW heterostructures, we now fabricate MoS_2_/NW-heterostructure-based photodetectors to benchmark them against the performance of bare MoS_2_ devices. The inset of Figure 5a shows an optical image of the fabricated devices. All phototransistors show n-type behavior in the transfer curve (see Figure S5a). The output characteristics of the devices under dark and 532 nm laser illumination conditions at a constant gate voltage (*V*_g_) of 20 V are shown in Figure 5a,b. The excitation polarization direction is always kept parallel to the NW axis during all the photocurrent measurements. Under laser illumination, the current density (*J*_d_) in the devices increases for a corresponding gradual increase in incident optical power. The calculated photocurrent (*I*_ph_) values, defined as *I*_light_ – *I*_dark_, as a function of laser power in a range of 50–500 μW are presented in Figure 5c. Saturated *I*_ph_ curves are observed in both types of phototransistors. In comparison with the bare MoS_2_ photodetector, we observe ∼5 and ∼8 times enhancement in *I*_ph_ from the MoS_2_/NW device at a laser power of 50 μW and 500 μW, respectively. This can be attributed to the strong EM field confinement at the top of NW that reinforces the applied electric field in the MoS_2_ channel to pass more photogenerated carriers toward the electrodes. For photodetectors, responsivity (*R*) and specific detectivity (D*) are two important parameters that give a measure of conversion efficiency and ability to detect weak optical signals, respectively:^51^
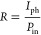
1
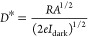
2where *P*_in_, *A*, and *e* are incident optical power, irradiation area, and the elementary charge of the electron, respectively. Figure 5d includes a comparison of calculated *R* and D* values at different laser powers. In general, the values of both parameters decrease with increasing laser power. At lower power, the higher responsivity in all devices results from a reduced recombination of the electrons as the trap states in MoS_2_ capture photoexcited electrons, which leads to a prolonged carrier lifetime.^52^ At higher power, a dominating carrier recombination rate results in lower responsivity. In comparison with bare MoS_2_, at 100 μW laser power we observe an ∼3 and ∼5 times increase in *R* and D*, respectively, from the MoS_2_/NW device.

**Figure 6:**
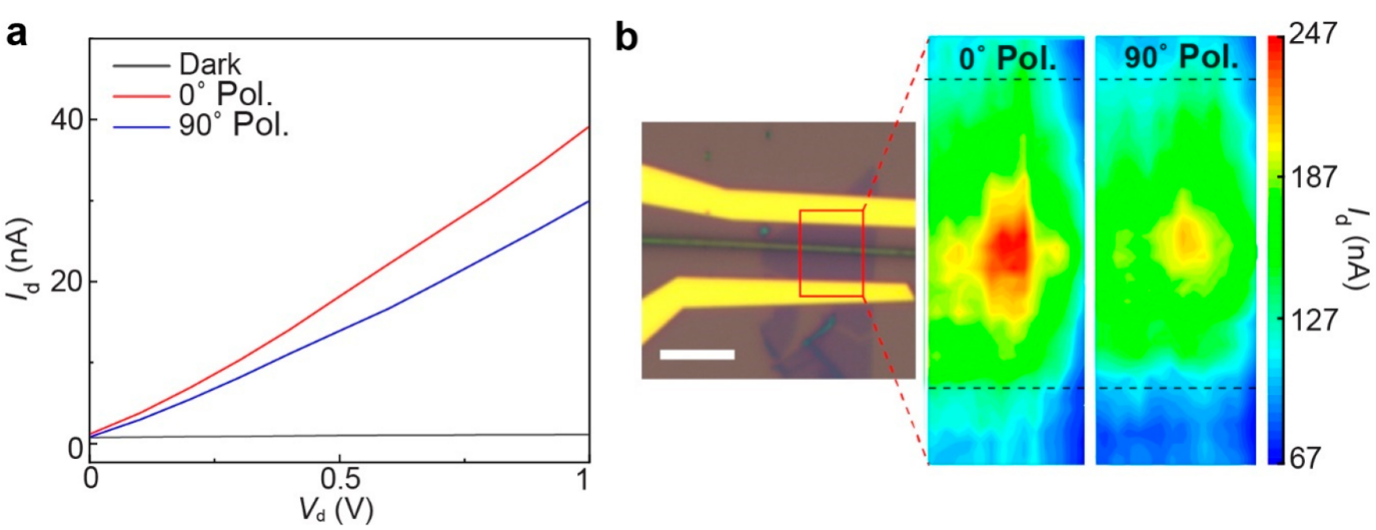
Anisotropy in the photoresponse of the mixed-dimensional MoS_2_/NW heterostructure. (a) Comparison of the *I*_d_ of the MoS_2_/NW device under dark conditions and under illumination of two different excitation polarizations. Polarizations at 0°and 90° correspond to excitation polarization parallel and perpendicular to the NW long axis, respectively, at *V*_g_ = 10 V. (b) Optical image of the device where the red rectangular box indicates the area of the photocurrent scan. Scale bar: 5 μm. The corresponding photocurrent maps with 0° and 90° excitation polarizations are shown on the right panel. The black dashed lines in the maps indicate the inner boundaries of the electrodes.

We study the dependence of photocurrent with linearly polarized light aligned parallelly (0°) and perpendicularly (90°) to the NW long axis to further understand the orientation of the maximum photocurrent in the MoS_2_/NW FET. As shown in Figure 6a, the maximum (∼36 nA) and minimum (∼27 nA) *I*_d_ are obtained with parallel and perpendicular polarization of the light, respectively, under the 532 nm laser with 100 μW power. The photocurrent mapping in Figure 6b further ensures the enhanced photocurrent along the NW, which varies with 0° and 90° polarization direction of the laser. This polarization-dependent photocurrent from MoS_2_ at the heterostructure is also attributed to the breaking of the rotational symmetry of monolayer MoS_2_ because of reduced dimensionality. Thus, an anisotropic photoresponse from isotropic 2D materials is achievable by virtue of 2D/1D mixed-dimensional heterostructures.

## Conclusions

We demonstrate enhancement and excitation polarization-sensitive optical and optoelectronic properties of monolayer MoS_2_ by integrating it with high refractive index AlGaAs NW. The Raman and PL intensity of MoS_2_ increases by ∼3 and ∼9 times in the MoS_2_/NW heterostructures compared with the bare MoS_2_ samples. Further, we observe a strong anisotropic response in the PL with an ∼60% degree of anisotropy because of the breaking of rotational symmetry induced by the NW. Numerical simulations reveal that excitation polarization-dependent EM field confinement of NWs is the basis of such optical responses. The mixed-dimensional photodetectors offer improved device performance with polarization sensitivity. In comparison with bare MoS_2_ phototransistors, the responsivity and specific detectivity in MoS_2_/NW devices increase by ∼3 and ∼5 times, respectively. Our results pave the way for 2D/1D mixed-dimensional heterostructure-based high-performance photonic and optoelectronic devices.

## Materials and Methods

### Sample Preparation

AlGaAs NWs are grown on a cleaned Si (111) substrate inside a horizontal flow atmospheric pressure metal–organic vapor phase epitaxy (MOVPE) system.^27,43^ NWs grown in this technique are predominantly zinc-blende phase. The cross-section of the NWs is assumed to be a hexagonal shape since most of the reported III–V semiconductor NWs grown along the [111] direction have a hexagonal crosssection.^53−55^ Afterward, as-grown NWs are transferred on top of the p-doped silicon substrate covered with a 285 nm thick SiO_2_ by nano combing technique.^43^ Monolayer and few-layer MoS_2_ crystals are grown on O_2_ plasma cleaned SiO_2_/Si substrate using a salt assisted CVD method.^56^ After a wet transfer process, the as-grown monolayer MoS_2_ flakes are transferred onto the NW nanocombed substrate. For hBN intercalated mixed-dimensional samples, commercially available MoS_2_ and hBN crystals (2D Semiconductors, USA) are used for exfoliation. All the samples are annealed at 250 °C for 2 h in a vacuum to ensure a clean interface.^57^

### Raman and PL Spectroscopy

The room-temperature Raman and PL spectra are collected in backscattering geometry with a confocal micro-Raman system (WITec alpha300 RA+). Samples are excited using a 532 nm laser with a spot size of less than 1 mm (×100 objective, 0.9 NA). Low laser power (<500 μW) is used to avoid laser-induced damage in the samples.

### Device Fabrication and Optoelectronic Measurements

After the material transfer, electron beam lithography (EBL Vistec, EPBG 5000) and metallization (MASA, IM-9912) are performed to realize Ti/Au (5/50 nm) electrodes. Optoelectronic measurements are carried out with a custom-built setup consisting of a confocal microscope (WITec Alpha 300 RA+) and two source meters (Keithley 2400 and 2401). During photomeasurements, samples are illuminated with ×20 [numerical aperture (NA) = 0.4] and ×100 (NA = 0.9) objective lenses to ensure proper illumination of the entire effective channel region.

### Theoretical Simulation

The numerical simulations are performed with the finite element modeling method using COMSOL Multiphysics. A hexagonal structure of 100 nm width with a 3.5 refractive index^50^ is placed on top of a 285 nm thick SiO_2_ layer, which is on top of a Si substrate. A linearly polarized plane wave is illuminated normally from the top to the bottom of the structure. The polar angle of this plane wave is varied from 0 to 360°; a polarization angle of 0 degrees corresponds to an EM field oscillating parallel to the NW, while a 90° angle corresponds to the perpendicular oscillation. Section S3 of the Supporting Information provides more information on simulations.

## Supporting Information Available

AFM characterization and NW diameter-dependent PL responses of the mixed-dimensional heterostructure; differential reflectivity spectra of the heterostructure, and PL response of a single AlGaAs NW; details of the simulation, transfer curves, and output *I*_d_*–V*_d_ curves of devices in dark condition; transfer curves of the devices under light illumination; photocurrent of the devices under different laser illuminations; and energy band alignment and charge transfer between MoS_2_ and AlGaAs NW (PDF)
