# Supplementary material for: Inducing Strong Light–Matter Coupling and Optical Anisotropy in Monolayer MoS2 with High Refractive Index Nanowire
Source: ACS Appl Mater Interfaces. 2022 Jun 28;14(27):31140–7. doi: 10.1021/acsami.2c07705 (PMC9284513; doi:10.1021/acsami.2c07705)
Supplement: Supplementary file 1 — am2c07705_si_001.pdf [file am2c07705_si_001.pdf]

Supporting Information

Inducing Strong Light–Matter Coupling and Optical Anisotropy in Monolayer MoS_2 with High Refractive Index Nanowire

Abde Mayeen Shafi,[†,] Faisal Ahmed,[†] Henry A. Fernandez,[†,‡] Md Gius Uddin,[†] Xiaoqi Cui,[†] Susobhan das,[†] Yunyun Dai,[†] Vladislav Khayrudinov,[†] Hoon Hahn Yoon,[†] Luojun Du,[†] Zhipei Sun,[†,‡] and Harri Lipsanen[†,*]*

[†]Department of Electronics and Nanoengineering, Aalto University, Tietotie 3, Espoo FI-02150, Finland

[‡]QTF Centre of Excellence, Department of Applied Physics, Aalto University, Aalto FI-00076, Finland

*Email: abde.shafi@aalto.fi, harri.lipsanen@aalto.fi

Table of contents:

S1. Effect of nanowire (NW) diameters on the optical response of MoS_2 ……………......….S3

S2. Optical characterizations of the samples ……………………………………………….S4

S3. Theoretical simulation details …………………………..……………….……….…..….S5

S4. Electronic response of the samples in dark ………………………….…………………..S7

S5. Incident optical power and wavelength-dependent electronic response of the samples ..S8

S6. Wavelength-dependent photocurrent of MoS_2 and MoS_2/NW devices ……….…..…....S9

S7. Band alignment in MoS_2 and AlGaAs NW …………...……………………………..….S9

References ………………………………..…………………………………….…………..…S10

Section S1: Effect of nanowire (NW) diameters on the optical response of MoS_2

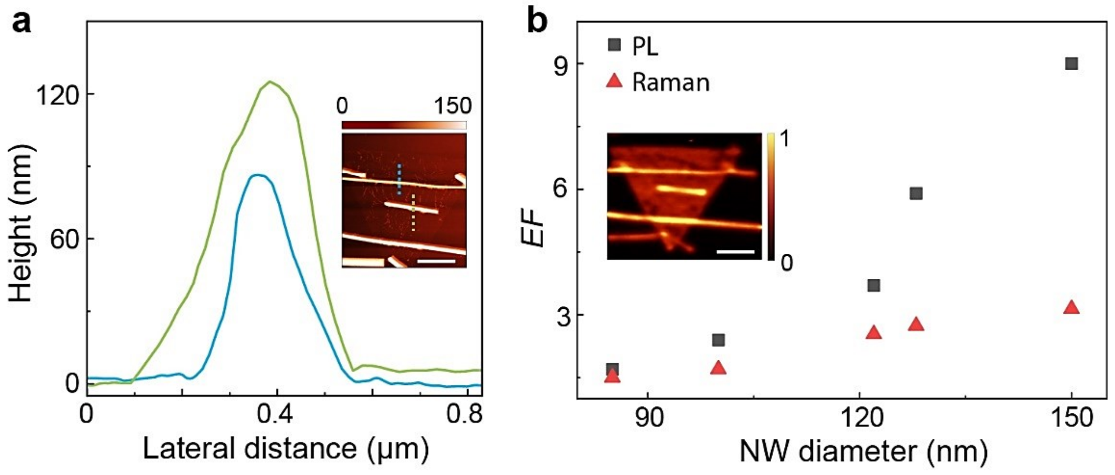

Figure S1. Morphology of a typical sample and effect of NW diameters on the optical response of MoS_2. **(a)** Atomic force microscopy (AFM) characterization of the mixed dimensional heterostructure sample. The diameter of the NWs in this sample typically ranges from 80 nm to 150 nm. Inset shows the AFM map with corresponding line scans. Scale bar: 2 µm. **(b)** NW diameter dependency of PL enhancement factor (*EF*). Inset shows the photoluminescent (PL) mapping of the MoS_2/NW heterostructure sample having NW of different diameters. Scale bar: 2 µm.

Strain calculation: The maximum uniaxial local strain induced by the NW on monolayer MoS_2 on the mixed-dimensional heterostructure is similar to the formation of MoS_2 wrinkle[1,2,3]. Strain ε can be estimated as,

$$\varepsilon \approx \frac{\pi^2 \delta h}{(1-\sigma^2)\lambda^2},$$

where δ, h, σ, and λ represent the MoS_2 thickness (~0.7 nm), the diameter of NW or height of curved MoS_2, Poison's ratio of MoS_2 (0.125)[1], and lateral distance of the curved MoS_2, respectively. For both 80 nm and 125 nm diameters of NW, the estimated local strain on monolayer MoS_2 is ~0.3%.

Section S2: Optical characterizations of the samples

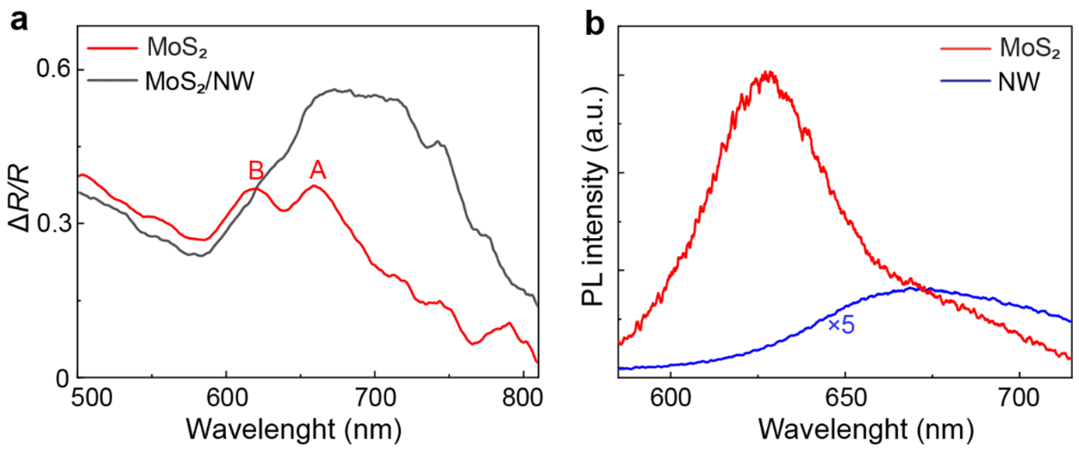

Figure S2. Linear optical responses from the samples. **(a)** Differential reflectivity spectra of MoS_2/NW heterostructure. Differential reflectivity[4] ($\Delta R/R$) is defined as $(R_{sample}-R_{substrate})/R_{substrate}$, where R_{sample} and $R_{substrate}$ are the reflection from the samples (MoS_2, MoS_2/NW) and the SiO_2 substrate, respectively. The peaks at 660 nm and 619 nm indicate A- and B- excitons of MoS_2, respectively. We can speculate from the differential reflectivity curve that the absorption is higher in the heterostructure region than bare MoS_2 due to high absorption in III-V semiconducting NWs[5]. **(b)** PL comparison from a typical AlGaAs NW and MoS_2 monolayer. The PL intensity of NW is multiplied by 5 for better comparison.

Section S3: Theoretical simulation details

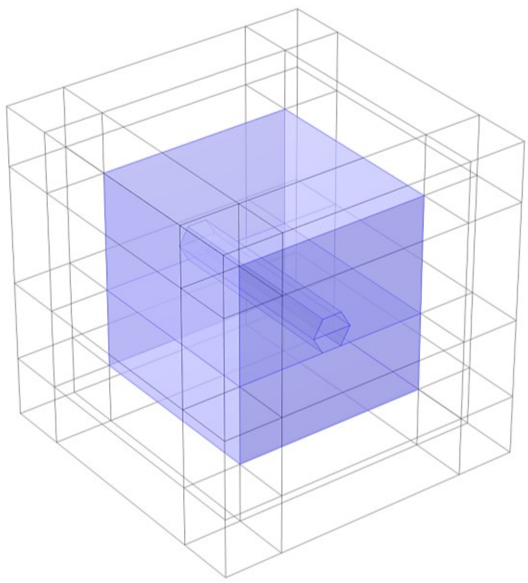

Figure S3. Three-dimensional geometry built-in COMSOL Multiphysics for the calculation of the scattered field by the hexagonal NW. In this geometry, a hexagonal NW is placed on top of the SiO_2/Si substrate. The refractive indices of the different materials are provided in the Materials and Methods section in the main text and Table 1. The calculations followed two steps: first, we calculate the full field of a propagating incident plane wave from the top of the geometry, without the NW. Second, we use the result from the first step as the incident field for calculating the scattered field by the NW. In the first step, we limit the geometry to the volume highlighted in the figure above, and we set the refractive index of the NW to be that of air. The plane wave is incident from a top port in this volume, and a bottom port is listening to the propagation. The other four vertical faces of the volume are set as a periodic boundary condition of the Floquet type. In the second step, the full field obtained from the first step is used as the background field for calculating the scattered field including the NW, now with a refractive index of $n = 3.5$. For this calculation, the whole volume shown in the figure above is used, with the surrounding domains set as perfectly matched layers. In this calculation, there are no listening ports, and there are no periodic boundary conditions. For the results presented in the main text, we take the values of the scattered field on the cross-section perpendicular to the NW, and in the center along the NW.

Heterostructure interface effect in theoretical analysis: We assume that MoS_2 and NW have flat surfaces and isotropic refractive indices during our calculation. The interface between the

different materials has tangential continuous boundary conditions for electromagnetic (EM) wave propagation, which neglects any electronic band-renormalization that might modify the optical properties of the heterostructure. Therefore, structural, electronic, or optical effects when bringing the materials in contact have not been considered in the FEM simulations. Table 1 presents a list of parameters used in the simulation.

Table 1: List of parameters used in the simulation.

**Parameters**	**Value**
Dimensions of NW	Diameter ~ 100 nm<br>Length ~ 5 µm
Thickness of MoS_2	0.7 nm
Thickness of SiO_2	285 nm
Refractive index (n)	NW ~ 3.5<br>MoS_2 ~ 3<br>Air ~ 1<br>SiO_2 ~ 1.45
Wavelength of incident light	532 nm

NW diameter-dependent EM field intensity: As shown in Figure S4, we observe oscillatory behaviour of the field intensity as a function of the NW diameter. This behaviour matches well with the previously reported results[6,7]. AlGaAs NW confines the electric field like in an optical cavity which implies that there are infinite confined modes, and their wavelengths depend on the effective NW diameter, which is d/n, where d is the NW diameter, and n is the refractive index. Our calculations show maxima at 87 nm and 140 nm. These maxima can be attributed to the first and second modes of the field confinement at 87 nm and 140 nm, respectively. These maxima as a function of the NW diameter might not correspond to the maxima of the PL as a function of the NW diameter, because our simulations are qualitative, and they might not reflect the real experimental conditions of the MoS_2/NW heterostructure. However, we think that the effect of the EM field confinement on MoS_2 PL would be much stronger when the MoS_2 interfacial contact area increases with increasing NW diameters. Therefore, the MoS_2 PL enhancement with an 80 nm diameter of NW is small even though the EM field intensity due

to confinement is high. On the contrary, the EM field intensity is moderately high at 150 nm diameter of NW but its effect on MoS_2 PL enhancement is extraordinarily strong due to the increase in the contact area of MoS_2 with NW.

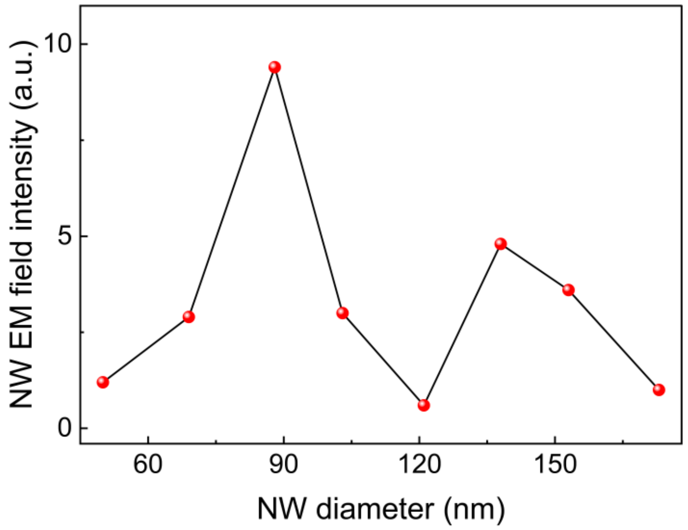

Figure S4: EM field intensity at the top of the NW upon plane wave illumination.

Section S4: Electronic response of the samples in dark

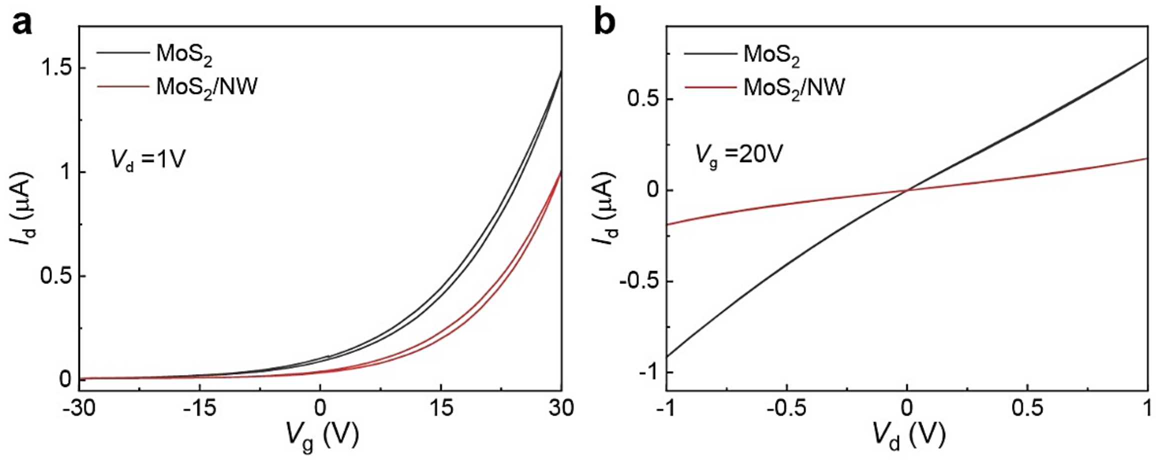

Figure S5. Electronic response of the devices. **(a)** Transfer characteristics of MoS_2 and MoS_2/NW devices in dark conditions at V_d = 1V. Devices show n-type transport property. The lower current in MoS_2/NW heterostructure (channel width ~1.15 µm) compared to bare MoS_2 (channel width ~1.94 µm) can be attributed to the larger channel width of the MoS_2 device. **(b)**

Output I_d-V_d characteristics of the same devices with constant V_g = 20 V. The graphs indicate high–quality contact between Ti/Au (5/50 nm) and channel materials.

Section S5: Incident optical power and wavelength-dependent electronic response of the samples

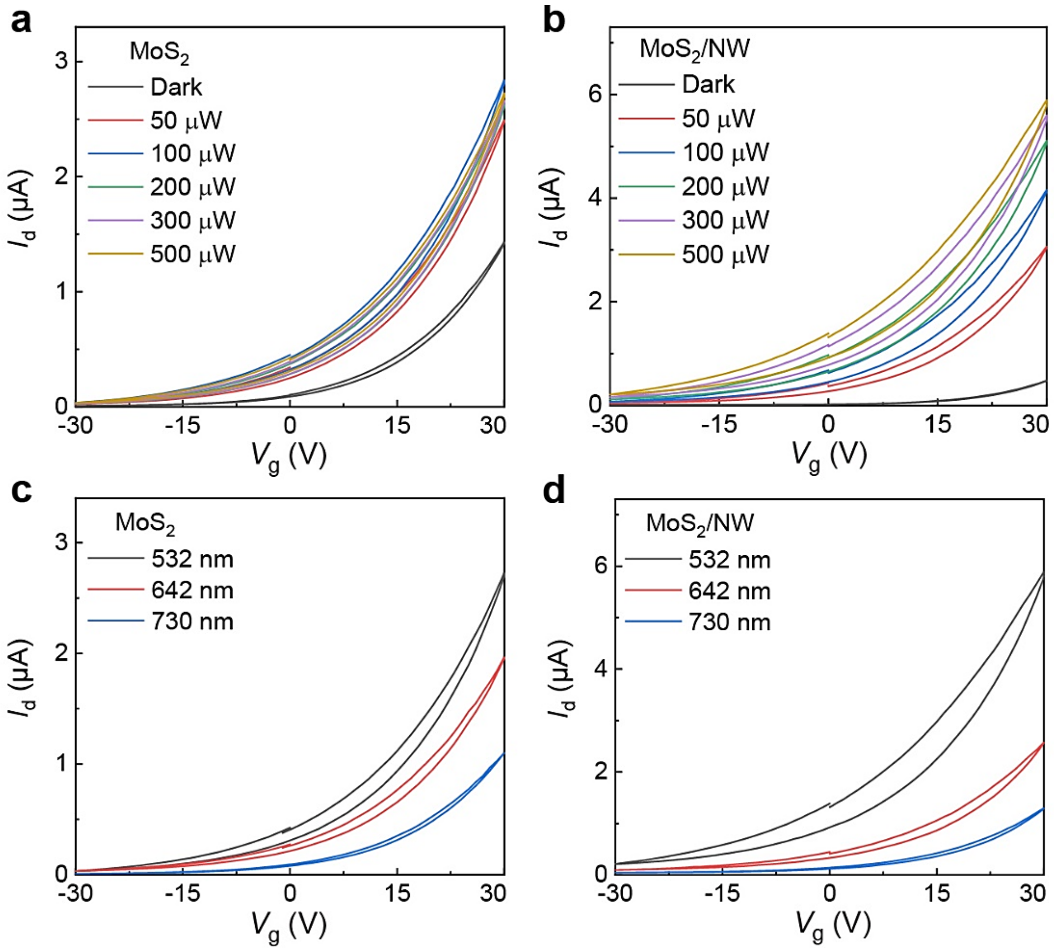

Figure S6. Transfer curves of the samples under illumination. **(a, b)** Transfer characteristics of MoS_2 and MoS_2/NW devices under dark and 532 nm laser illumination at V_d = 1V. The device current increases with increasing laser power. The maximum photocurrent in MoS_2/NW device is ~2 times higher than MoS_2. **(c, d)** Transfer characteristics of the devices under 532 nm, 642 nm, and 780 nm laser illuminations. A fixed laser power of 500 μW is used for all the photo-measurements. Device current decreases for lower energy excitations.

Section S6: Wavelength-dependent photocurrent of MoS_2 and MoS_2/NW devices

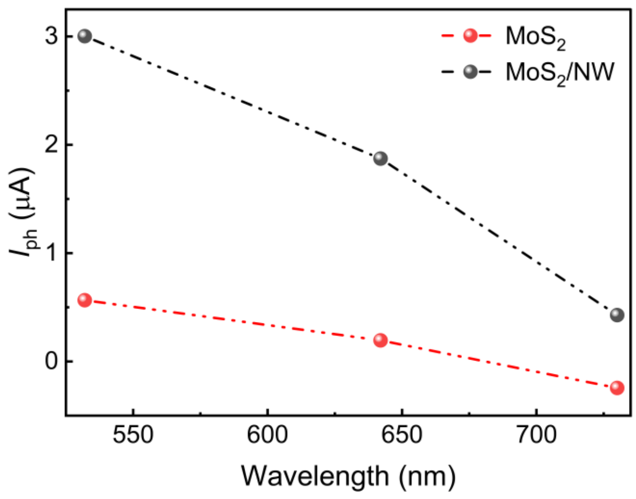

Figure S7. Photocurrent of MoS_2 and MoS_2/NW devices as a function of wavelength. Photocurrent decreases with the decreasing laser energy. The current is always higher in heterostructure than in bare MoS_2 devices.

Section S7: Band alignment in MoS_2 and AlGaAs NW heterostructure

When MoS_2 and AlGaAs come in physical contact, the energy bands in AlGaAs move upwards and in MoS_2 drop down to reach a thermal equilibrium state, thus resulting in a typical type-II band alignment at the interface, as shown in Fig. S8b. Owing to the Fermi level position difference, the build-in potential is induced across the interface between MoS_2 and AlGaAs NW. Under laser illumination with photon energy greater than the bandgap of individual materials, the photo-induced carriers are generated. The built-in electric field at the interface can separate and transport the photogenerated electron-hole carriers depending on band alignment position. Under zero bias conditions, the hole carriers from p-doped AlGaAs can be transported to the MoS_2, while electrons from MoS_2 face a larger barrier at the interface to surmount the barrier. That's why in Fig. S5(a), a slight p-doping trend is observed for heterostructure as compared to only MoS_2.

From the band alignment, we also observe that the charge transfer between these heterostructure materials is very weak. This results in the influence of NW field confinement on optical properties of MoS_2 being more dominant over the charge transfer. Based on this analysis, we assume that this mixed-dimensional heterostructure method is applicable to enhance the optical properties of other TMDC monolayers if the band alignment of the heterostructure materials induces weak charge redistribution.

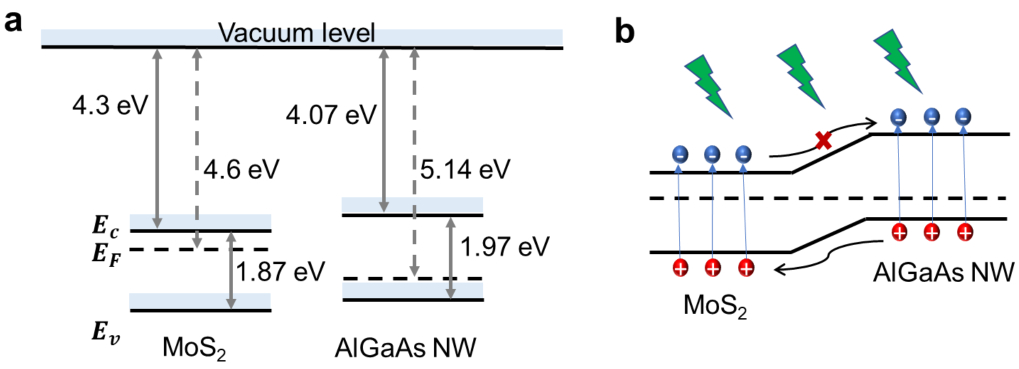

Figure S8. Energy band alignment and charge transfer between MoS_2 and AlGaAs NW. **(a)** The band position of monolayer MoS_2 and AlGaAs NW before contact. **(b)** The carrier transport between MoS_2 and AlGaAs.

References

1. Castellanos-Gomez, A.; Roldán, R., Cappelluti, E.; Buscema, M.; Guinea, F.; van der Zant, H.S.; Steele, G.A. Local Strain Engineering in Atomically Thin MoS_2. *Nano lett.* **2013**, *13*(11), 5361-5366.

2. Luo, S.; Hao, G.; Fan, Y.; Kou, L.; He, C.; Qi, X.; Tang, C.; Li, J.; Huang, K.; Zhong, J. Formation of Ripples in Atomically Thin MoS_2 and Local Strain Engineering of Electrostatic Properties. *Nanotechnology* **2015**, *26*(10), 105705.

3. Vella, D.; Bico, J.; Boudaoud, A.; Roman, B.; Reis, P. M. The Macroscopic Delamination of Thin Films from Elastic Substrates. *Proc. Natl. Acad. Sci. U. S. A.*

2009, *106*, 10901–10906.

4. Ruppert, C.; Aslan, O. B.; Heinz, T. F. Optical Properties and Band Gap of Single- and Few-Layer $MoTe_2$ Crystals. *Nano Lett.* **2014**, *14*, 6231–6236.

5. Liu, L.; Diao, Y.; Xia, S. Enhanced Optical Absorption of Gradient-component $Al_xGa_{1-x}As$ Nanowire Arrays for Solar Cell Applications. *Opt. Commun.* **2020**, *477*, 126340.

6. Khorasaninejad, M.; Dhindsa, N.; Walia, J.; Patchett, S.; Saini, S. S. Highly Enhanced Raman Scattering from Coupled Vertical Silicon Nanowire Arrays. *Appl. Phys. Lett.* **2012**, *101*, 173114.

7. Notomi, M.; Takiguchi, M.; Sergent, S.; Zhang, G.; Sumikura, H. Nanowire Photonics toward Wide Wavelength Range and Subwavelength Confinement. *Opt. Mater. Express* **2020**, *10*, 2560.
